# System-Dependent Ecotoxicological Effects of Anatase and Rutile Titanium Dioxide Nanoparticles Across Prokaryotic and Eukaryotic Test Models

**DOI:** 10.3390/nano16140849

**Published:** 2026-07-10

**Authors:** Gergely Krett, Rózsa Farkas, Máté Varga, Tamás Annus, Linda Marzougui, Ádám Solti, Károly Bóka, Erika Tóth

**Affiliations:** 1 Department of Microbiology, Eötvös Loránd University, Pázmány Péter Lane 1/c, 1117 Budapest, Hungary; 2 Department of Genetics, Eötvös Loránd University, Pázmány Péter Lane 1/c, 1117 Budapest, Hungary; 3 Department of Plant Physiology and Molecular Plant Biology, Eötvös Loránd University, Pázmány Péter Lane 1/c, 1117 Budapest, Hungaryadam.solti@ttk.elte.hu (Á.S.); 4 Department of Plant Anatomy, Eötvös Loránd University, Pázmány Péter Lane 1/c, 1117 Budapest, Hungary; karoly.boka@ttk.elte.hu; 5 Research and Development Center, Eszterházy Károly Catholic University, Leányka Str. 8/G, 3300 Eger, Hungary

**Keywords:** TiO_2_ nanoparticles, rutile, anatase, toxicity, microbial communities, plant bioassay, zebrafish embryo developmental assay, genotoxicity

## Abstract

The use of nanoscale materials has expanded rapidly in recent decades, with titanium dioxide (TiO_2_) nanoparticles among the most widely produced. Their increasing environmental release raises concerns about ecosystem-level effects. A key mechanism of toxicity is the generation of reactive oxygen species (ROS), although these effects strongly depend on particle properties, including crystalline form, size, morphology, surface characteristics, and exposure conditions. Here, we assessed and compared the ecological impacts of anatase and rutile TiO_2_ nanoparticles across prokaryotic and eukaryotic systems, including aquatic microbial communities, microbial cultures, plant bioassays (*Sinapis alba*, *Triticum aestivum*), the SOS Chromotest, and zebrafish (*Danio rerio*) assays. Nano-TiO_2_ exposure markedly restructured freshwater microbial communities by suppressing sensitive taxa (e.g., Actinomycetota, *Flavobacterium*, and *Limnohabitans*) while enriching more tolerant genera such as *Pseudomonas*, *Sediminibacterium*, *Haliscomenobacter*, and *Hydrogenophaga*. These shifts likely reflect differences in cell-envelope structure, biofilm formation, and antioxidant capacity. The two investigated TiO_2_ nanoparticle types showed distinct biological effects: rutile was associated with more pronounced microbial community shifts and bacterial cell damage, whereas anatase caused stronger responses in plant assays, particularly by impairing hypocotyl growth and plant water balance. Besides the limited acute genotoxicity revealed by the SOS Chromotest, TiO_2_ nanoparticles did not significantly affect survival or final larval body length in the zebrafish embryo assay under the tested conditions; however, the hatching delay observed at the highest concentration indicated a sublethal developmental effect. Overall, our results show that TiO_2_ nanoparticle toxicity cannot be generalized across biological systems and suggest that biological responses depend on the combined influence of particle characteristics and organism-specific structural and physiological traits.

## 1. Introduction

The application of nanoscale materials has increased rapidly in recent decades across diverse fields. Their defining feature—particle sizes between 1 and 100 nm—confers unique properties, largely because of their high surface area-to-volume ratio. Nanoparticles may be derived from both inorganic and organic materials (e.g., metals, metal oxides, silicates, carbon, and ceramics), and they exhibit physical, chemical, electrical, mechanical, and optical characteristics that differ from those of their bulk counterparts [1]. These properties are strongly influenced by particle size and structure.

Titanium dioxide (TiO_2_) nanoparticles are among the most widely produced and utilized nanomaterials [2]. They are commonly used as white pigments in paints and cosmetics and are also applied in the pharmaceutical and electronics industries. In the European Union, TiO_2_ (E171) was permitted as a food additive until 2022 under Regulation (EC) No. 1333/2008 [3] but was subsequently banned because of concerns regarding potential toxicity and genotoxicity (Commission Regulation (EU) 2022/63; Commission Implementing Regulation (EU) 2021/2090) [4,5]. In the United States, the Food and Drug Administration is currently reviewing its regulatory status, while JECFA concluded that there was no safety concern for the evaluated flavouring substances [6]. Despite these restrictions, TiO_2_ nanoparticles remain in use, including in food packaging materials [7].

Titanium dioxide is a chemically stable, optically active, and widely applied metal oxide nanomaterial whose properties are strongly determined by crystal phase, particle size, morphology, surface chemistry, and synthesis history [8]. The most commonly discussed TiO_2_ polymorphs are anatase, rutile, and brookite [9]. Although these phases share the same chemical composition, their different crystal structures result in distinct physicochemical behavior, including differences in density, optical response, electronic structure, phase stability, surface reactivity, and photocatalytic activity [10]. Rutile is generally considered the thermodynamically most stable TiO_2_ phase, whereas anatase and brookite are metastable and may transform into rutile during thermal treatment, although this phase stability is also influenced by nanoscale dimensions, dopants, and surface-energy effects [9]. These structural differences are relevant not only for technological applications but also for biological and environmental interactions, because photocatalytic activity, ROS generation, aggregation behaviour, and surface reactivity can all influence nanoparticle toxicity.

Nano-TiO_2_ can be produced by several routes, including sol–gel, hydrothermal, extraction–pyrolytic, precipitation, thermal, and green-synthesis approaches. These methods affect the final properties of the material, including crystal phase composition, crystallite size, particle morphology, aggregation state, surface hydroxylation, impurities, and dopant distribution [11]. Consequently, the biological effects of TiO_2_ nanoparticles cannot be interpreted solely on the basis of chemical composition. Proper characterization commonly requires complementary techniques, such as TEM or SEM for morphology and particle size, X-ray diffraction and Raman spectroscopy for crystalline phase identification, EDX for elemental composition, XPS for surface chemical states, BET analysis for specific surface area, and DLS and zeta-potential measurements for colloidal behavior in suspension [11,12]. Such physicochemical parameters are particularly important in ecotoxicological studies, because they may influence nanoparticle mobility, sedimentation, cell-surface interactions, photocatalytic activity, and ROS-mediated effects. Therefore, the comparison of anatase and rutile TiO_2_ nanoparticles provides a useful framework for investigating how differences in particle properties are associated with biological responses across different organisms.

Owing to their large-scale production, nano-TiO_2_ particles are increasingly released into the environment, where they may affect ecosystem functioning. A primary mechanism of toxicity involves the generation of reactive oxygen species (ROS), leading to lipid peroxidation, membrane damage, and increased permeability. These effects can impair mitochondrial function, inhibit oxidative phosphorylation, and reduce cellular viability [13]. Elevated ROS levels are further associated with apoptosis, necrosis, fibrosis, genotoxicity, inflammation, and other adverse outcomes [14], and neurotoxic effects have also been reported [15,16].

However, TiO_2_ nanoparticle toxicity is not determined by ROS generation alone, but depends on the combined effects of particle characteristics, exposure conditions, and organism-specific sensitivity [13,17,18]. Many studies do not distinguish clearly between different TiO_2_ forms or particle properties, and direct comparisons between anatase and rutile remain relatively limited.

Given the widespread use, environmental release, and documented biological effects of TiO_2_ nanoparticles, this study aimed to assess the ecological impacts of different crystalline forms on prokaryotic and eukaryotic systems. Because TiO_2_ nanoparticles accumulate in aquatic environments [19], exposure of these systems is particularly relevant [20]. Accordingly, the effects of anatase and rutile nanoparticles were evaluated using aquatic microcosms, microbial cultures, plant bioassays (*Sinapis alba* and *Triticum aestivum*), the SOS Chromotest, and zebrafish (*Danio rerio*) assays.

## 2. Materials and Methods

### 2.1. Preparation, Application, and Verification of Titanium Dioxide Solutions

Aqueous colloidal dispersions of anatase and rutile TiO_2_ nanoparticles (NPs) were provided by Holikem Ltd. (Budapest, Hungary). The form and size of the nanoparticles were verified by transmission electron microscopy. The diameter of the isometric spherical anatase particles (CAS No: 1317-80-2) was 15.8 ± 1.7 nm on average, while the elongated, rod-shaped rutile particles (CAS No: 1317-80-2) had a length of 46.7 ± 2.2 nm and a diameter of 13.7 ± 0.7 nm. Both crystal types were monomorphic, with no detectable presence of other forms. The TiO_2_ nanoparticle preparations were characterized based on supplier information and the data reported by Geiszelhardt et al. (2025) [16], where the same TiO_2_ dispersions and preparation procedure were applied in a parallel study conducted in cooperation with our research group. The available characterization included crystal form, morphology, and primary particle size. Additional morphometric analysis connected to the mineral forms of the TiO_2_ were not determined.

The concentration range of nano TiO_2_ applied in the present study was selected to include both lower exposure levels closer to reported environmental and hotspot concentrations and higher concentrations suitable for mechanistic toxicity assessment. Reported environmental concentrations of engineered TiO_2_ nanoparticles in surface waters are generally expected to occur in the ng/L to low µg/L range, although higher values may occur locally and transiently in waters affected by wastewater discharge, urban runoff, sewage spills, recreational activities, or industrial inputs. The highest concentrations applied in the experiments therefore should not be interpreted as representative of typical natural-water exposure, but rather as high-dose treatments used to evaluate concentration-dependent biological responses and to compare the relative effects of anatase and rutile TiO_2_ nanoparticles.

### 2.2. Microcosm Experiments

Sampling for the microcosm experiments was carried out in two runs from a temperate river impacted by urban settlements, on the right shore of the Danube River in Budapest, Hungary (47.477357° N; 19.061891° E). Water was collected into a 1.5 L clean, sterile, screw-capped glass bottle. The samples were transported to the laboratory at 4 °C and used for TiO_2_ exposure experiments on prokaryotic communities in laboratory microcosms.

During preparation of the TiO_2_ solutions, 200 mL aliquots of water sample were dispensed into microcosm systems (250 mL clean, sterile glass bottles), and TiO_2_ was added to final concentrations of 50, 100, 200, 500, 1000, 1800, 2500, and 3500 ppm. Separate dilution series were prepared for the rutile and anatase crystal forms. The assembled microcosms were shaken at 80 rpm in a Witeg Wis10 shaking incubator (Witeg, Wertheim, Germany) for five weeks at 23 °C.

The bacterial community compositions of the microcosm samples were assessed by high-throughput amplicon sequencing. Community DNA was isolated from the microcosms as described by Toumi et al. [21]. PCR amplification was performed in triplicate in a final volume of 20 μL containing 1× Phusion HF Buffer (Thermo Fisher Scientific Inc., Waltham, MA, USA), 0.2 mM dNTPs (Fermentas, Vilnius, Lithuania), 0.4 μg μL^−1^ bovine serum albumin (Fermentas), 0.3 μM of each primer, and 0.4 U Phusion High-Fidelity DNA Polymerase (Thermo Fisher Inc.). The V3–V4 region of the 16S rRNA gene was amplified using CS1-TS-B341F (5′-CCT ACG GGN GGC WGC AG-3′) [22] and modified CS2-TS-806RM primers (5′-GGA CTA CHV GRG TWT CTA AT-3′) [23], with the following thermal conditions: initial denaturation at 98 °C for 5 min, followed by 25 amplification cycles of 30 s at 95 °C, 30 s at 55 °C, and 30 s at 72 °C, followed by a final extension at 72 °C for 10 min. Library processing and amplicon sequencing were performed on an Illumina MiSeq platform (Illumina Inc., San Diego, CA, USA) at the Genomics Core Facility RTSF, Michigan State University, USA, as detailed by Szuróczki et al. [24]. The resulting sequence reads were analysed using Mothur software [25] following the MiSeq SOP pipeline. Taxonomic affiliation was assigned using the ARB-SILVA SSU NR reference database, release 138_1 [26]. Operational taxonomic units (OTUs) were assigned at a 97% similarity threshold [27]. Raw sequence reads were deposited in the NCBI SRA database and are accessible under BioProject ID PRJNA1442401.

### 2.3. Selective Cultivation of Bacteria from Microcosm Systems Treated with TiO_2_ Nanomaterials

Bacterial strains were isolated from the microcosm containing 200 ppm of the anatase form of TiO_2_. Isolation was performed using the standard dilution plate technique on DSM medium 1 (https://www.dsmz.de/microorganisms/medium/pdf/DSMZ_Medium1.pdf, accessed on 25 June 2026) supplemented with 200 ppm TiO_2_. Incubation was carried out at 28 °C for seven days. DNA was extracted from the isolated bacterial strains [28], and PCR amplification of the 16S rRNA gene was then performed using the primers 27F (5′-AGA GTT TGA TCM TGG CTC AG-3′) and 1492R (5′-GGT TAC CTT GTT ACG ACT T-3′) [29]. The 16S rRNA gene sequencing was carried out at LGC (Berlin, Germany). The sequenced bacterial strains were identified using the EzBioCloud online identification system (accessed on 25 June 2026) [30], and the sequences were submitted to the NCBI GenBank database under accession numbers PZ178833–PZ178839.

Two of the cultivated bacterial strains were selected to test their sensitivity to both TiO_2_ nanomaterial forms (anatase and rutile). As a first step, an agar diffusion assay [31] was performed with increasing concentrations of the two nanomaterial forms (50, 200, 500, 1650, 2500, and 5000 ppm) to determine the minimal concentration that was effective against the bacterial strains. The original bacterial cell concentration was 10^8^ CFU mL^−1^. After treatment, TEM studies were performed using a Hitachi 7100 transmission electron microscope [32] to examine the effects on cell morphology. Finally, 1650 ppm of rutile or anatase was used to treat the bacterial strains as follows: the number of cells in nutrient medium (DSM1) was adjusted to 10^7^ CFU mL^−1^. The number of living cells was determined regularly on days 2, 6, 9, and 15 after nanomaterial treatment.

### 2.4. Testing Phytotoxicity

The impact of TiO_2_ nanoparticles on germination and seedling growth was tested using the white mustard (*Sinapis alba* L.) germination test. Seeds were surface-sterilized with 30% H_2_O_2_ for 10 min. To test germination, seeds were placed on filter papers moistened with 0.5 mM CaSO_4_ supplemented with TiO_2_ nanoparticles in a dose range of 5 to 2500 ppm and incubated in darkness for three days. Germination percentage and radicle and hypocotyl lengths were evaluated.

Phytotoxicity experiments were performed on winter bread wheat (*Triticum aestivum* L. cv. MV Nemere) plants. Surface-sterilized seeds were germinated on filter papers moistened with 0.5 mM CaSO_4_ under light for three days. Germinated kernels were transferred to stainless-steel grids in beakers and cultivated in 400 mL of 1/4-strength Hoagland solution (1.25 mM Ca(NO_3_)_2_, 1.25 mM KNO_3_, 0.5 mM MgSO_4_, 0.25 mM KH_2_PO_4_, 0.08 μM CuSO_4_, 4.6 μM MnCl_2_, 0.19 μM ZnSO_4_, 0.12 μM Na_2_MoO_4_, 11.56 μM H_3_BO_3_, and 10 μM Fe(III)-EDTA) in a growth chamber with 12/12 h light (150 µmol m^−2^ s^−1^)/dark periods, 20/18 °C temperature, and 70/75% relative humidity. The nutrient solution was refreshed weekly. Plants were treated from the three-leaf stage with the two crystalline forms of TiO_2_ nanomaterials at 50, 500, and 2500 ppm, based on effective doses identified in preliminary studies, for two weeks. During treatment, the nutrient solution was not refreshed but was stirred daily and adjusted to a volume of 400 mL at the end of the first week with 1/4-strength Hoagland solution.

To measure stomatal conductance reflecting leaf transpiration, a Delta-T porometer (Delta-T Devices Ltd., Cambridge, UK) was applied. Measurements were performed on the abaxial side of the middle sections of the third leaves, which had partially developed under nanomaterial exposure. Chlorophyll contents were determined in 80% (*v*/*v*) acetone buffered with 5 mM Tricine (pH 8.0) extracts using a UV-VIS spectrophotometer (Shimadzu 2100, Kyoto, Japan) and the absorption coefficients of Porra et al. [33]. Relative water content (RWC) of the root system and foliage was measured by recording fresh weight (FW) after harvesting. The nutrient solution was removed from the root system by centrifugation for 2 min at 800 rpm in a filter-paper sandwich. Saturated weight (SW) was measured after overnight incubation on wet filter paper in a humid environment. Dry weight (DW) was measured after desiccation at 60 °C until air-dry. RWC was calculated as RWC = (FW − DW)/(SW − DW) × 100.

### 2.5. Ecotoxicological Investigations

#### 2.5.1. SOS Chromotest-Based Ecotoxicological Investigations

The ecotoxicity of different concentrations (640, 320, 160, 80, 40, 20, 10, and 5 ppm) and crystal forms (anatase and rutile) of nano-TiO_2_ was assessed using the SOS Chromotest (EBPI, Burlington, ON, Canada) according to the manufacturer’s instructions. Higher concentrations could not be tested because the method is based on optical density measurements and, at higher concentrations, the nanoparticles covered the surface of the wells of the ELISA plates. The SOS induction factor (SOSIF), representing the genotoxicity of the investigated compound, was calculated as described in the manual provided by the manufacturer.

#### 2.5.2. TiO_2_ Nanoparticle Toxicity on Zebrafish Development

Experiments were conducted on wild-type larvae of the AB strain. Adult AB fish were maintained in the fish facility of ELTE Eötvös Loránd University under standard conditions [34,35]. Embryos were raised in E3 medium (5 mM NaCl, 0.17 mM KCl, 0.33 mM CaCl_2_, and 0.33 mM MgSO_4_ in distilled water) supplemented with methylene blue. During the experiments, hatching rate, survival, and length were analysed after treatment with anatase and rutile forms of TiO_2_ at concentrations of 10, 100, and 1000 ppm. For the toxicity tests, zebrafish were treated from 4 h post-fertilization (hpf) to 5 days post-fertilization (dpf) (*n* = 20 per treatment). For hatching rate analysis, treatments were performed in duplicate and hatched specimens were counted daily. For the survival assay, non-dechorionated zebrafish were treated and dead specimens were counted daily. Total larval length (in pixels) after TiO_2_ treatment was measured at 5 dpf in Fiji [36]. Length analysis was also carried out under light exposure. In this case, larvae were initially raised in darkness (using an unlit incubator) at 28.5 °C. At 32 hpf, larvae were removed from the incubator and kept at room temperature to expose them to ambient sunlight in the laboratory. Following nano-TiO_2_ treatments, lengths were measured at 4 hpf.

All experiments were carried out in accordance with the Hungarian Act of Animal Care and Experimentation (1998, XXVIII) [37] and Directive 2010/63/EU of the European Parliament and of the Council of 22 September 2010 [38] on the protection of animals used for scientific purposes. All protocols and experimental procedures used in this work were approved by the Hungarian National Food Chain Safety Office (PEI/001/1713-2/2015) and the ELTE Eötvös Loránd University, Faculty of Science Animal Welfare Commission.

### 2.6. Statistical Analysis

For visualization of the microbial community data by multivariate statistical analysis, R software v.4.4.2 (https://www.R-project.org/, accessed on 25 June 2026) and the vegan package [39] were used. Significantly different sample groups were identified using one-way PERMANOVA, homogeneity of multivariate dispersion was assessed using a multivariate dispersion test, and the contribution of taxa to the observed dissimilarities was determined by SIMPER analysis; all analyses were performed using PAST v.5.3 software [40].

In the germination assays, 40 seeds per treatment were investigated in three replicates. In the plant physiological toxicity assays, three plant individuals per treatment were exposed to TiO_2_ nanoparticles in two independent repetitions. Porometry was performed in three technical repetitions on leaves at the same developmental stage from all plant individuals. Chlorophyll measurements were performed on mid-leaf sections of the leaves used for porometry. In the fresh and dry weight measurements, plant individuals were handled separately. Student’s *t*-tests were performed to compare differences between two data groups. One-way ANOVA with Tukey–Kramer post hoc analyses was performed in GraphPad Prism v.9.2 (GraphPad Software Inc., Boston, MA, USA) to compare variance among multiple leaf groups. The term “significantly different” refers to *p* < 0.05.

## 3. Results

### 3.1. Bacterial Community Responses in Microcosms

**Figure 1:**
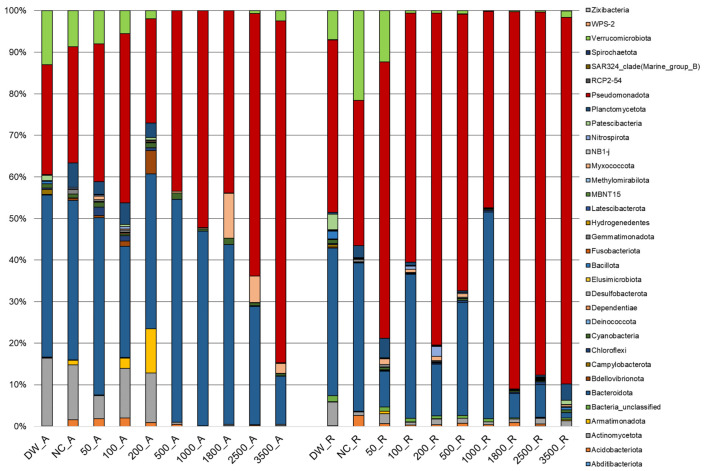
Bacterial community compositions of the microcosms treated with different concentrations of TiO_2_ nanoparticles at the phylum level. In the sample names, the numbers represent the ppm concentration of TiO_2_ nanoparticles in the microcosms, and the following A or R indicates anatase or rutile crystal form, respectively. DW refers to the native freshwater sample from the Danube River (Danube water), and NC indicates the negative control without treatment, which was incubated along with the treated samples.

After quality filtering and chimera removal, a total of 402,456 amplicon reads were identified and affiliated with 33 bacterial phyla. At this taxonomic rank, the bacterial community compositions of samples treated with different crystal types of nano-TiO_2_ differed significantly from each other. Furthermore, samples incubated with rutile differed significantly from the phylum composition of the control samples according to one-way PERMANOVA. Regarding changes in the abundance of dominant phyla, Pseudomonadota-related sequences became more dominant at higher nanoparticle concentrations for both crystal types. In contrast, the abundance of Bacteroidota, Actinomycetota, and Verrucomicrobiota decreased with increasing TiO_2_ concentrations (Figure 1).

**Figure 2:**
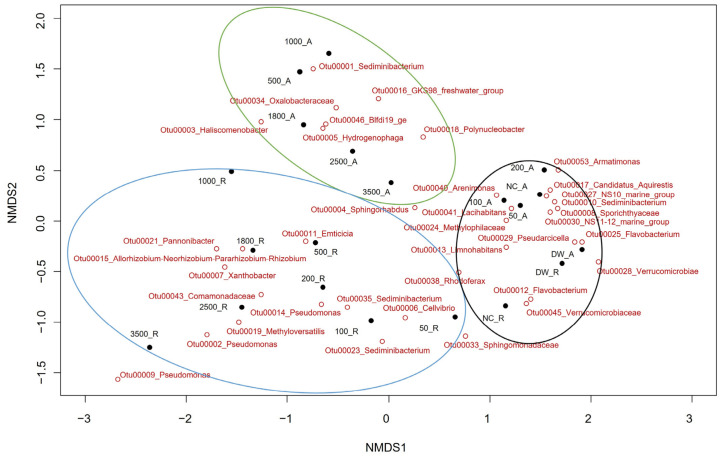
OTU-based NMDS plot of the bacterial community compositions of the microcosms treated with different TiO_2_ nanoparticle concentrations. Only OTUs contributing up to 70% cumulative dissimilarity are shown. Circles indicate significantly different (*p* < 0.05) groups (blue: rutile at high and low concentrations; green: anatase at high concentrations; black: controls and anatase at low concentrations). In the sample names, the numbers represent the ppm concentration of TiO_2_ nanoparticles in the microcosm, and the following A or R indicates anatase or rutile crystal form, respectively. DW refers to the native freshwater sample from the Danube River (Danube water) without incubation or treatment, and NC indicates the negative control without treatment, which was incubated along with the treated samples. Stress = 0.17.

Considering OTU-based results from the bacterial community investigations, significant differences were observed between the taxonomic composition of samples treated with rutile and the negative controls, as well as between rutile- and anatase-treated samples. By contrast, among anatase-treated microcosms, only those treated with higher concentrations (>200 ppm) showed significant alterations compared with the community compositions of the negative controls (Figure 2). However, it should be noted that the significant multivariate dispersion test indicated differences in within-group heterogeneity among treatments. Therefore, the PERMANOVA results should be interpreted with caution, as the detected differences may reflect not only shifts in community composition, but also differences in variability among samples. Some of the most abundant OTUs identified from the fresh, untreated, and unincubated Danube water (DW) communities and from the untreated but incubated control microcosms (NC) belonged to typical freshwater bacteria inhabiting lakes and riverine habitats, such as *Flavobacterium*, *Limnohabitans*, and *Rhodoferax*, indicating a relatively unbiased bacterial community even after incubation of the untreated microcosms. Similar community compositions were observed in microcosms treated with anatase nano-TiO_2_ below 500 ppm. At higher anatase concentrations, *Sediminibacterium*-, *Haliscomenobacter*-, and *Hydrogenophaga*-related sequences comprised the most abundant OTUs. However, significant differences were observed compared with the initial and untreated freshwater communities even at low rutile concentrations. At higher concentrations, *Pseudomonas*-related sequences were the most dominant and were represented by three different OTUs. In addition to this genus, members of *Xanthobacter* and *Allorhizobium*-*Neorhizobium*-*Pararhizobium*-*Rhizobium* were also identified as dominant taxa, suggesting possible tolerance to high concentrations of rutile.

### 3.2. Cultivation-Based Microbiological Investigations

Using a selective cultivation approach on DSM 1 medium containing 200 ppm TiO_2_, seven bacterial strains were identified and affiliated with four species of the genus *Pseudomonas*. Four strains showed the highest sequence similarity to *Pseudomonas alcaligenes*, while the closest relatives of the remaining three were *P. vancouverensis*, *P. mandelii*, and *P. koreensis*. Two strains—*Pseudomonas alcaligenes* (T2) and *Pseudomonas vancouverensis* (T5)—were used to perform agar diffusion assays with different concentrations and two crystal forms of nano-TiO_2_. An inhibitory effect occurred only above 500 ppm for rutile and 1650 ppm for anatase in both strains, and the effect was weak, with 10–12 mm inhibition zones observed.

**Figure 3:**
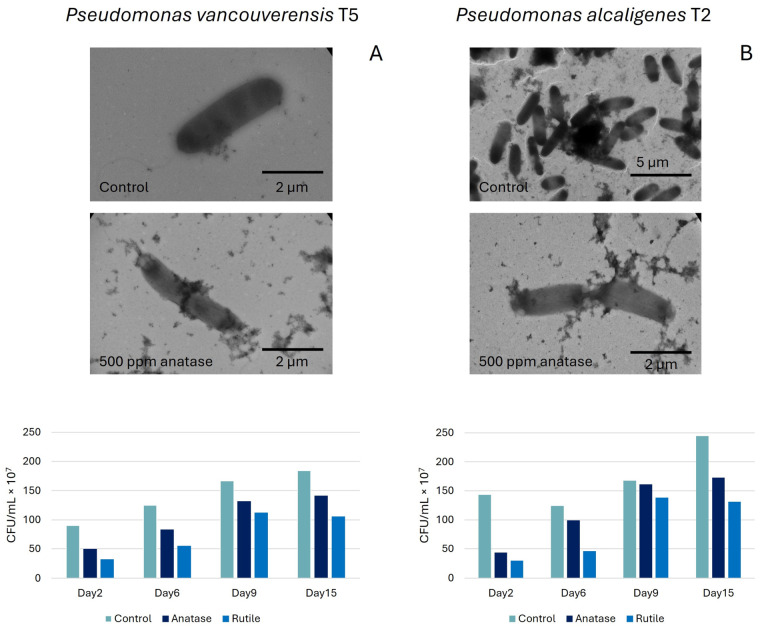
Effect of TiO_2_ nanoparticles on the multiplication of *Pseudomonas vancouverensis* T5 (**A**) and *Pseudomonas alcaligenes* T2 (**B**), and changes in their abundance over time after 1650 ppm nano-TiO_2_ treatment.

TEM results showed that the lower concentrations (50–200 ppm) of the nanomaterials did not affect cell morphology. In TEM images, multiplication disorders appeared mainly in both *P. alcaligenes* and *P. vancouverensis* strains after rutile treatment at 500 ppm, affecting 10–15% of cells (Figure 3). As the 1650 ppm concentration appeared to be effective against both bacterial strains for both nanomaterial forms, the final growth test was performed using this concentration. Our results show that 1650 ppm nanomaterial treatment influenced the multiplication of both bacterial strains. Rutile appeared to be more effective, but after nine days, the differences in CFU values were not significant compared with the control (Figure 3).

### 3.3. Genotoxicity Assessment by SOS Chromotest

**Figure 4:**
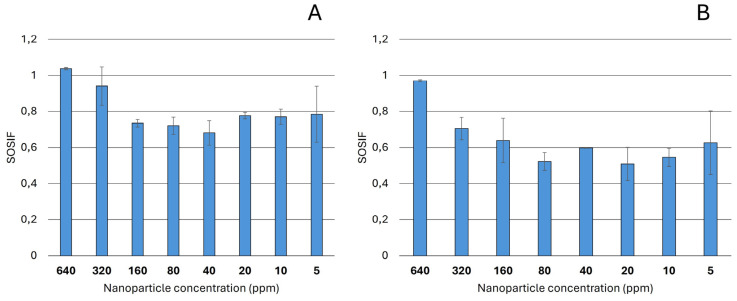
SOSIF values of different crystal forms and concentrations of TiO_2_ nanoparticles ((**A**): anatase, (**B**): rutile; error bars represent SD values).

According to the SOSIF values obtained by the SOS Chromotest, no clear genotoxic effect (SOSIF > 2) of TiO_2_ nanoparticles was observed in the investigated concentration range (Figure 4). However, elevated SOSIF values were detected at the highest TiO_2_ nanoparticle concentration for both crystal forms.

### 3.4. Phytotoxicity of TiO_2_ Nanoparticles

**Figure 5:**
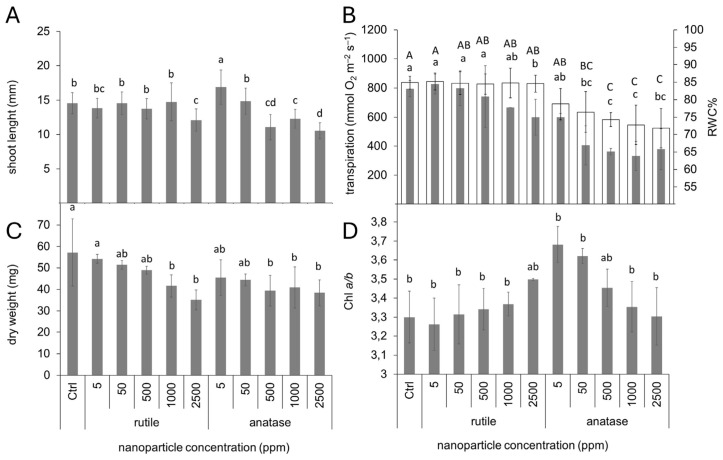
(**A**) Shoot (hypocotyl) elongation of white mustard seedlings in the germination assay exposed to multiple concentrations of rutile and anatase TiO_2_ nanoparticles (*n* = 21–39). (**B**) Relative water content (RWC%; open columns; statistical groups in capital letters) and transpiration (grey columns; statistical groups in lowercase letters); (**C**) shoot dry weight; and (**D**) chlorophyll (Chl) a/b ratio in the second leaves of wheat plants cultivated hydroponically in the presence of rutile and anatase TiO_2_ nanoparticles (*n* = 5 in (**B**–**D**)). Error bars represent SD values. To compare differences among stages, one-way ANOVAs were performed with Tukey–Kramer post hoc tests (*p* < 0.05).

The effects of TiO_2_ nanoparticles were tested on germinating seeds of white mustard. Although exposure to up to 2500 ppm TiO_2_ nanoparticles affected neither germination percentage (average germination percentage: 97.5 ± 11.5%; one-way ANOVA, *p* = 0.900; variation among treatments was not significant) nor root growth (24.6 ± 11.7 mm; one-way ANOVA, *p* = 0.762; variation among treatments was not significant), shoot growth was affected. Under rutile exposure, shoot elongation was affected only at 2500 ppm, whereas anatase exposure resulted in a gradual decline in shoot elongation with increasing nanoparticle concentrations (Figure 5A).

Phytotoxicity of TiO_2_ nanoparticles was also tested in a hydroponic wheat model. Neither anatase nor rutile TiO_2_ nanoparticles induced significant variation in root dry weight (23.7 ± 5.6 mg; one-way ANOVA, *p* = 0.285; variation among treatments was not significant). However, the treatments significantly affected shoot physiology (Figure 5B,C). While exposure to rutile nanoparticles resulted in no alterations in shoot relative water content (Figure 5B) and caused decreases in transpiration rate and shoot dry weight only at higher concentrations (Figure 5B,C, respectively), anatase nanoparticles caused larger, concentration-dependent decreases in all these parameters. The lowest applied concentration of anatase nanoparticles (5 ppm) already caused significant decreases in all these parameters compared with the untreated control (Ctrl). Although none of the TiO_2_ nanoparticle crystalline forms or concentrations decreased the total leaf chlorophyll content on a fresh-weight basis (1670 ± 272 µg chlorophyll a + b mg^−1^ fresh weight; one-way ANOVA, *p* = 0.213; variation among treatments was not significant), a significant alteration was measured in the leaf chlorophyll a/b ratio (Figure 5D). Exposure to increasing concentrations of rutile nanoparticles induced only a slight increase in the chlorophyll a/b ratio toward the higher concentrations, whereas anatase nanoparticles resulted in a significant increase in the chlorophyll a/b ratio at low concentrations but smaller or non-significant alterations at higher concentrations.

### 3.5. Effects of TiO_2_ Nanoparticles on Zebrafish Development

**Figure 6:**
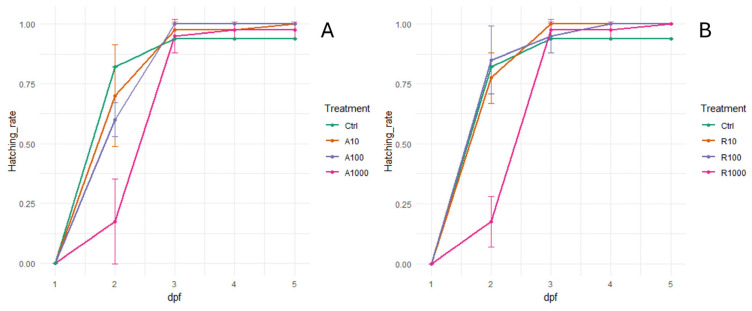
Hatching rate of zebrafish embryos treated with different concentrations of nano-TiO_2_ ((**A**): anatase, (**B**): rutile; Ctrl: control, dpf: days post-fertilization).

Overall hatching success of zebrafish embryos was not affected by nanoparticle treatments up to 100 ppm; however, larvae exposed to 1000 ppm of either anatase or rutile showed an initial hatching delay (Figure 6). By contrast, anatase and rutile exposure had no significant effect on survival during the first five days of development, and overall median length was not affected by treatments either with or without sunlight exposure (Supplementary Figures S1–S3).

## 4. Discussion

In agreement with our findings, several studies have demonstrated that TiO_2_ nanoparticles can significantly alter the composition of freshwater bacterial communities, as the sensitivity of different taxa to nano-TiO_2_ varies considerably [18,41]. An investigation conducted in Lake Michigan and the Chicago River showed that the relative abundance of typical freshwater taxa, such as members of Actinomycetales and the genera *Flavobacterium* and *Limnohabitans*, decreased following nanoparticle exposure, while others, including *Limnobacter*, became more abundant, potentially affecting both community functionality and stability [15]. Consistent with these observations, two of the most abundant OTUs in our untreated control microcosms—affiliated with *Flavobacterium* and *Limnohabitans*—were absent from microcosms containing high nanoparticle concentrations (Figure 2). Similarly, members of the phylum Actinomycetota were significantly more abundant in control communities or in those exposed to low nanoparticle concentrations, further supporting the sensitivity of these taxa to TiO_2_ toxicity. At the same time, previous work has shown that nano-TiO_2_ may differentially affect prokaryotic and eukaryotic primary producers. Chen et al. [41] reported that eukaryotic algae were more sensitive to nano-TiO_2_ exposure than members of Cyanobacteriota, and network analyses indicated substantial nanoparticle-driven restructuring of eukaryotic microbial communities. Elevated tolerance of cyanobacterial taxa was also observed in the present study. Although their initial abundance in Danube water samples was low (~1%), similar or even higher relative abundances were detected in some high-concentration treatments (e.g., 1.5% at 1800 ppm anatase), suggesting resilience to TiO_2_ nanoparticle exposure (Figure 1). Physiological traits likely play a key role in determining taxon-specific tolerance. Cell-wall structure has been proposed as an important determinant of sensitivity [42]. In comparative studies, Gram-positive *Bacillus subtilis* exhibited greater membrane permeability following nano-TiO_2_ exposure than Gram-negative *Escherichia coli* and *Pseudomonas aeruginosa*, implying increased vulnerability of Gram-positive cell envelopes. This may partly explain the decline in Actinomycetota observed under high nanoparticle concentrations. Additional protective mechanisms may further contribute to differential survival. Several studies have shown that exposure to TiO_2_ nanoparticles can induce exopolysaccharide (EPS) production, which may mitigate toxicity by binding nanoparticles and limiting their interaction with cell surfaces [43,44]. Although EPS production and biofilm formation were not quantified in the present study and their involvement should therefore be considered hypothetical, these mechanisms may help explain the dominance of EPS-producing genera such as *Pseudomonas* in microcosms exposed to high rutile concentrations. Members of this genus are well known for their biofilm-forming capacity and EPS production, traits that are associated with enhanced resistance to environmental stressors [45]. Similarly, protection against reactive oxygen species (ROS) generated by nano-TiO_2_ may represent another possible survival strategy. Antioxidant enzymes such as superoxide dismutase, catalase, and peroxidases can neutralize ROS and reduce oxidative damage. However, because ROS production, antioxidant enzyme activity, lipid peroxidation, and other oxidative stress biomarkers were not directly measured in the present study, the involvement of oxidative stress remains hypothetical. Nevertheless, in silico genome analyses and biochemical characterization of cultivated *Pseudomonas* species revealed the presence of multiple antioxidant defence systems, which may contribute to their tolerance under TiO_2_ nanoparticle exposure (https://bacdive.dsmz.de/strain/13123; https://bacdive.dsmz.de/strain/12807; accessed on 25 June 2026). Furthermore, time-dependent toxicity experiments indicated that such detoxification mechanisms likely require induction, as differences in CFU counts between treated and untreated samples diminished after nine days (Figure 3). Other dominant taxa observed under high nanoparticle exposure—including *Xanthobacter*, *Rhizobium*, and *Hydrogenophaga*—may similarly benefit from EPS-mediated protection [46,47,48]. Structural adaptations may also play a role. For instance, the sheath-forming *Haliscomenobacter*-related bacterium may gain protection from its extracellular sheath, while the enrichment of *Sediminibacterium* under anatase exposure is consistent with previous observations of its persistence on TiO_2_-containing remediation materials [49,50]. Beyond EPS production and antioxidant defences, bacteria may employ additional strategies, such as modifying nanoparticle surfaces, altering cell-surface charge, or restructuring membrane composition, to reduce interactions with nanoparticles, including ROS-mediated effects and direct physical damage caused by nanotubes or needle-like nanocrystals (e.g., rutile). Such adaptive changes have been reported in *Escherichia coli*, *Ochrobactrum*, and *Pseudomonas putida* exposed to various nanomaterials [51,52], and may contribute to the observed dominance of *Pseudomonas* in rutile-treated systems. Importantly, our results also revealed marked differences between the effects of anatase and rutile forms of nano-TiO_2_. In our microbial systems, rutile treatment was associated with more pronounced shifts in community composition at lower concentrations and caused a higher frequency of defective cells, as observed by TEM. Higher-resolution TEM images further supported the presence of TiO_2_ nanoparticles in close association with the surface of *Pseudomonas* cells after both anatase and rutile exposure (Supplementary Figure S4). Division abnormalities were frequently observed in cells exposed to both nanoforms. Anatase particles were often observed attached to the cell surface, whereas the elongated rutile particles appeared to be in close contact with the cell envelope and, in some cases, were associated with local deformation or apparent penetration-like interactions. However, these observations are qualitative and do not allow quantitative evaluation of nanoparticle uptake or definitive intracellular localization. These findings are consistent with previous reports indicating greater ROS generation by rutile than by anatase [53], as well as studies demonstrating stronger impacts of rutile on gut microbial communities [54]. Taken together, these findings suggest that nanoparticle toxicity is highly context-dependent and influenced by multiple factors, including particle size, morphology, aggregation state, surface chemistry, exposure conditions, and organism-specific physiological traits. Although our results indicate greater microbial toxicity of rutile nanoparticles, caution is warranted when extrapolating these effects across biological systems. Differences in uptake pathways, cellular architecture, and redox regulation may fundamentally shape responses to distinct TiO_2_ crystalline forms, as also reflected by divergent responses reported in plant systems.

Similarly to our findings, previous studies directly comparing anatase and rutile TiO_2_ nanoparticles have reported partly contradictory toxicity patterns, indicating that the biological effects of these materials cannot be generalized across systems. Anatase TiO_2_ has often been described as more reactive or more toxic than rutile, mainly because of its higher photocatalytic activity and ROS-generating potential. For example, Jin et al. [55] reported that anatase TiO_2_ nanoparticles generated ROS more readily than rutile after dispersion. In addition to crystal phase, particle morphology may also influence toxicity. Tong et al. [56] suggested that the higher toxicity of spherical anatase particles could partly result from more favourable particle–cell contact geometry, allowing closer interaction with the cell surface. However, direct contact is not necessarily required for toxicity, as Planchon et al. [57] showed that nano-TiO_2_ may exert adverse effects even without intimate particle–cell contact. Moreover, Lin et al. [58] emphasized that particle size, ionic strength, and pH strongly influence the toxicity of anatase TiO_2_ toward *E. coli*, highlighting the importance of exposure conditions. In contrast, several studies have reported stronger effects of rutile TiO_2_ nanoparticles. Yu et al. [59] found higher toxicity of rutile than anatase TiO_2_ nanoparticles in macrophages despite comparable particle size, surface area, and zeta potential, suggesting that differences in affinity to proteins and phospholipids, rather than ROS generation alone, may contribute to toxicity. Similarly, Uboldi et al. [60] reported that rutile TiO_2_ induced stronger cytotoxicity, genotoxicity, and morphological transformation than anatase in fibroblast cells. Phase-specific but endpoint-dependent effects were also observed by Iswarya et al. [61], who investigated anatase and rutile TiO_2_ nanoparticles in *Chlorella* sp. and found that the two crystal forms caused distinct types of cellular damage: anatase primarily affected the nucleus and cell membrane, whereas rutile induced damage to chloroplasts and internal organelles. Interestingly, mixtures of anatase and rutile have also been shown to produce non-uniform responses. Depending on concentration and phase ratio, combined exposure may result in either antagonistic or additive toxicity compared with the individual crystal forms. Gerloff et al. [62] similarly reported that anatase/rutile-containing TiO_2_ samples were more toxic to human intestinal cells than pure anatase samples; however, the expression of ROS-responsive genes was not increased, suggesting that mechanisms other than oxidative stress may also be involved. These contradictory findings suggest that TiO_2_ nanoparticle toxicity is governed by the combined effects of crystal phase, particle size, morphology, aggregation behaviour, surface properties, exposure medium, illumination conditions, and the biological endpoint investigated. Therefore, the higher microbial impact of rutile observed in the present study should not be interpreted as a universal feature of this crystal form, but rather as a system-dependent response arising from the interaction between rutile particle morphology, microbial cell-surface properties, and exposure conditions. Conversely, the stronger effects of anatase in our plant assays may reflect different uptake barriers, nanoparticle mobility, tissue-level sensitivity, and physiological endpoints.

In contrast to the effects of TiO_2_ nanomaterial crystalline forms on microbial communities and bacterial isolates, anatase caused stronger responses in the plant models. Although neither anatase nor rutile affected germination, anatase affected hypocotyl elongation at higher concentrations. The effects of TiO_2_ nanoparticles on plants appear to be highly taxon-specific, as Song et al. [63] found no inhibition, but rather enhancement, of seed germination and seedling growth in tomato (*Lycopersicum esculentum*) exposed to an anatase:rutile 80:20 mixture (27 nm particle size) in the 50–5000 ppm range. Because root growth of germinating seeds was not affected in our study, whereas hypocotyl growth was affected, the effect appears to be indirect. Moreover, the direct effect of rutile nanoparticles, which severely affected bacterial cells, seemed to be attenuated in plants. We presume that this difference may arise from differences in cell-wall structure and surface area-to-volume ratio between plants and bacteria. In our experiments with wheat plants grown hydroponically, rutile was also less toxic than anatase. In the case of anatase, disruption of the shoot water regime appeared to be pivotal, as indicated by decreasing RWC% and transpiration. The increase in the chlorophyll a/b ratio under low-dose anatase treatment indicated suppressed biosynthesis of chlorophyll b-containing antennae. However, together with decreased growth, this effect on the photosynthetic apparatus was not evident when total chlorophyll content was measured on a fresh-weight basis. The lower chlorophyll a/b ratio at higher anatase concentrations suggests that, in addition to antennae, the accumulation of reaction centres was also affected. Du et al. [64] reported that approximately 100 ppm TiO_2_ (particle size < 100 nm) applied to wheat under soil conditions resulted in Ti-containing agglomerate formation around the root periderm, and the nanoparticles also adhered to the cell walls. Jacob et al. [65] postulated that iron plaque in roots could also be an important factor in titanium retention. Kelemen et al. [66] also found that Ti ascorbate treatment of wheat grown hydroponically resulted in retention of the majority of Ti in the roots. However, the Ti translocated to the shoot was not associated with the cell wall and thus represented a soluble Ti fraction. Nevertheless, both Ti mobilization and translocation of entire TiO_2_ nanoparticles are possible in plants, although experimental conditions can profoundly influence these effects [67]. Although our study did not distinguish ionic Ti from nanoparticulate Ti translocation, the higher toxicity of anatase in our system is suggested to be connected with the smaller size and larger surface area-to-volume ratio of anatase nanoparticles compared with rutile nanoparticles. Consequently, unlike in bacteria, where direct cellular damage and ROS generation appear to be primary effects, Ti mobilization and/or TiO_2_ nanoparticle mobility may be principal factors driving toxicity in plants in aquatic systems or solution-based exposure settings.

Exposure of zebrafish embryos to TiO_2_ nanoparticles resulted in concentration-dependent developmental effects, indicating that fish development, similarly to plant hypocotyl growth, was affected at elevated nanoparticle concentrations. Previous studies have shown that TiO_2_ nanoparticles can negatively affect zebrafish embryo development and survival, particularly after dechorionation [68]. Increased incidence of stunted larval growth has also been reported, especially under concurrent illumination [63]. In the present study, overall hatching success and survival during the first five days of development were not significantly affected by either anatase or rutile treatment, which is consistent with previous reports showing limited effects on these apical endpoints [69,70]. This limited effect may partly be explained by the protective role of the chorion during early embryonic development. However, exposure to the highest tested concentration of both rutile and anatase TiO_2_ nanoparticles (1000 ppm) caused a significant delay in hatching, demonstrating that TiO_2_ nanoparticles can interfere with pre-hatching developmental progression even in the absence of marked lethality. In contrast, Samaee et al. [71] reported a shortened time to hatching after nano-TiO_2_ exposure, further supporting that TiO_2_ nanoparticles may affect pre-hatching development, although the direction and magnitude of this response appear to depend on exposure conditions, particle properties, and the developmental endpoints investigated. Therefore, our zebrafish results should be interpreted as evidence of sublethal developmental effects on the measured apical endpoints rather than as a general assessment of developmental toxicity. Additional morphological, physiological, behavioral, or molecular endpoints would be required to fully characterize the developmental impact of TiO_2_ nanoparticles in zebrafish embryos.

In addition to direct physical effects on cells, TiO_2_ nanoparticle toxicity may also involve molecular biological impacts. Genotoxicity of nano-TiO_2_ in eukaryotic cells has previously been investigated mainly by comet and micronucleus assays, indicating that nanoparticles can induce genotoxic damage [72,73,74,75]. Regarding the mechanism of action, Bhattacharya et al. [76] found that TiO_2_ nanoparticles can generate free radicals that may cause DNA adduct formation and eventually lead to permanent mutations and changes in transcription. However, other studies have indicated no DNA-damaging effect when prokaryotic and eukaryotic cells were incubated in the presence of nanoparticles [77,78,79]. Based on the limited number of studies using the SOS Chromotest, nano-TiO_2_ has been considered to have no or weak genotoxic effects at lower concentrations (0–100 ppm), although findings have been highly inconsistent [80,81]. Our results showed that neither rutile nor anatase nano-TiO_2_ was genotoxic within the investigated concentration range (5–640 ppm), but the increasing SOSIF values observed for both forms at higher concentrations suggest a possible DNA-damaging effect at high concentrations.

Overall, these findings support the view that TiO_2_ nanoparticle effects are not determined by crystal phase alone, but arise from the combined influence of particle properties, exposure conditions, and organism-specific structural and physiological traits.

## 5. Conclusions

Overall, our results show that TiO_2_ nanoparticles can exert taxon-specific and system-dependent biological effects across the investigated microbial, plant, and animal models. In freshwater microbial communities, exposure to nano-TiO_2_ reshaped community composition by reducing several sensitive taxa, including members of the phylum Actinomycetota and the genera *Flavobacterium* and *Limnohabitans*, while favouring bacteria such as *Pseudomonas*, *Sediminibacterium*, *Haliscomenobacter*, and *Hydrogenophaga*. These shifts may be related to differences in physiological traits, including cell-envelope structure, biofilm formation, and antioxidant defence capacity. The two investigated TiO_2_ nanoparticle types showed distinct biological effects, suggesting that particle properties, including crystalline form, size, and morphology, contribute to toxicity in a system-dependent manner. Rutile treatment was associated with more pronounced effects on microbial communities and cultivated bacterial cells, possibly reflecting the combined influence of particle shape, surface interactions, ROS-mediated stress, and direct cellular damage. In contrast, anatase treatment caused stronger responses in plant assays, particularly by affecting hypocotyl growth and plant water balance, suggesting that nanoparticle mobility, surface reactivity, and plant-specific uptake or barrier properties may be important determinants of phytotoxicity. In zebrafish embryos, TiO_2_ nanoparticles did not significantly affect survival or final larval body length under the tested conditions, but hatching delay at the highest concentration indicates a sublethal developmental effect on the measured apical endpoints. However, to fully characterize the developmental impact of TiO_2_ nanoparticles on zebrafish, additional morphological, physiological, behavioral, or molecular endpoints would be required. Taken together, our findings indicate that the toxicity of TiO_2_ nanoparticles cannot be generalized across biological systems or attributed to crystal structure alone. Instead, biological responses appear to depend on the combined effects of particle characteristics and organism-specific structural and physiological traits. These results emphasize the importance of context-dependent risk assessment when evaluating the environmental impacts of engineered nanomaterials.

## Data Availability

The data presented in this study are available in the article and Supplementary Materials. Raw amplicon sequence reads have been deposited in the NCBI SRA database under BioProject accession number PRJNA1442401. The 16S rRNA gene sequences of the isolated bacterial strains have been deposited in the NCBI GenBank database under accession numbers PZ178833–PZ178839.

## Supplementary Materials

The following supporting information can be downloaded at https://www.mdpi.com/article/10.3390/nano16140849/s1. Figure S1: Overall survival probability of zebrafish embryos treated with different concentrations of nano-TiO_2_ (A: anatase, B: rutile, Ctrl: control). Figure S2: Total length of zebrafish embryos treated with different concentrations of nano-TiO_2_ in the absence of light measured at 96 hours post-fertilization (A: anatase, B: rutile, Ctrl: control, D: dark, L: light). Figure S3: Total length of zebrafish embryos treated with different concentrations of nano-TiO_2_ in the presence of sunlight measured at 5 days post-fertilization (A: anatase, R: rutile, Ctrl: control). Figure S4: Transmission electron microscopy images of *Pseudomonas vancouverensis* strain T5 after treatment with 500 ppm anatase (A) and rutile (B) TiO_2_ nanoparticles. TiO_2_ nanoparticles were observed in close association with the bacterial cell surface.

## References

[1] Roy D., Adhikari S., Adhikari A., Ghosh S., Azahar I., Basuli D., Hossain Z. (2022). Impact of CuO nanoparticles on maize: Comparison with CuO bulk particles with special reference to oxidative stress damages and antioxidant defense status. Chemosphere.

[2] Bundschuh M., Filser J., Lüderwald S., McKee M.S., Metreveli G., Schaumann G.E., Schulz R. (2018). Nanoparticles in the environment: Where do we come from, where do we go to?. Environ. Sci. Eur..

[3] (2008). Regulation (EC) No. 1333/2008 of the European Parliament and of the Council of 16 December 2008 on Food Additives.

[4] (2022). Commission Regulation (EU) 2022/63 of 14 January 2022 Amending Annexes II and III to Regulation (EC) No. 1333/2008 of the European Parliament and of the Council as Regards the Food Additive Titanium Dioxide (E 171).

[5] (2021). Commission Implementing Regulation (EU) 2021/2090 of 25 November 2021 Concerning the Denial of Authorisation of Titanium Dioxide as a Feed Additive for All Animal Species.

[6] JECFA (2023). Summary Report of the 97th Meeting.

[7] Li J., Zhang D., Hou C. (2024). Application of nano-titanium dioxide in food antibacterial packaging materials. Bioengineering.

[8] Hsu C.-Y., Mahmoud Z.H., Abdullaev S., Ali F.K., Naeem Y.A., Mizher R.M., Mizher R.M., Karim M.M., Abdulwahid A.S., Ahmadi Z. (2024). Nano titanium oxide (nano-TiO_2_): A review of synthesis methods, properties, and applications. Case Stud. Chem. Environ. Eng..

[9] Manzoli M., Freyria F.S., Blangetti N., Bonelli B. (2022). Brookite, a sometimes under evaluated TiO_2_ polymorph. RSC Adv..

[10] Eddy D.R., Permana M.D., Sakti L.K., Sheha G.A.N., Solihudin, Hidayat S., Takei T., Kumada N., Rahayu I. (2023). Heterophase polymorph of TiO_2_ (anatase, rutile, brookite, TiO_2_(B)) for efficient photocatalyst: Fabrication and activity. Nanomaterials.

[11] Podelinska A., Neilande E., Pankratova V., Serga V., Bandarenka H., Burko A., Piskunov S., Pankratov V.A., Sarakovskis A., Popov A.I. (2025). Structural and spectroscopic characterization of TiO_2_ nanocrystalline materials synthesized by different methods. Nanomaterials.

[12] Ji Z., Jin X., George S., Xia T., Meng H., Wang X., Suarez E., Zhang H., Hoek E.M., Godwin H. (2010). Dispersion and stability optimization of TiO_2_ nanoparticles in cell culture media. Environ. Sci. Technol..

[13] Erdem A., Metzler D., Cha D.K., Huang C.P. (2015). The short-term toxic effects of TiO_2_ nanoparticles toward bacteria through viability, cellular respiration, and lipid peroxidation. Environ. Sci. Pollut. Res..

[14] Yu Z., Li Q., Wang J., Yu Y., Wang Y., Zhou Q., Li P. (2020). Reactive oxygen species-related nanoparticle toxicity in the biomedical field. Nanoscale Res. Lett..

[15] Song B., Zhang Y., Liu J., Feng X., Zhou T., Shao L. (2016). Unraveling the Neurotoxicity of Titanium Dioxide Nanoparticles: Focusing on Molecular Mechanisms. Beilstein J. Nanotechnol..

[16] Geiszelhardt E., Tóth E., Bóka K., Bencsik N., Schlett K., Tárnok K. (2025). Glia Cells Are Selectively Sensitive to Nanosized Titanium Dioxide Mineral Forms. Int. J. Mol. Sci..

[17] Battin T.J., Kammer F.V., Weilhartner A., Ottofuelling S., Hofmann T. (2009). Nanostructured TiO_2_: Transport behavior and effects on aquatic microbial communities under environmental conditions. Environ. Sci. Technol..

[18] Binh C.T.T., Tong T., Gaillard J.F., Gray K.A., Kelly J.J. (2014). Acute effects of TiO_2_ nanomaterials on the viability and taxonomic composition of aquatic bacterial communities assessed via high-throughput screening and next generation sequencing. PLoS ONE.

[19] Asztemborska M., Jakubiak M., Stęborowski R., Chajduk E., Bystrzejewska-Piotrowska G. (2018). Titanium dioxide nanoparticle circulation in an aquatic ecosystem. Water Air Soil Pollut..

[20] Singh S., Prasad S.M., Bashri G. (2023). Fate and toxicity of nanoparticles in aquatic systems. Acta Geochim..

[21] Toumi M., Abbaszade G., Sbaoui Y., Farkas R., Ács É., Jurecska L., Tóth E. (2021). Cultivation and molecular studies to reveal the microbial communities of Groundwaters discharge located in Hungary. Water.

[22] Herlemann D.P.R., Labrenz M., Jürgens K., Bertilsson S., Waniek J.J., Andersson A.F. (2011). Transitions in bacterial communities along the 2000 km salinity gradient of the Baltic Sea. ISME J..

[23] Kozich J.J., Westcott S.L., Baxter N.T., Highlander S.K., Schloss P.D. (2013). Development of a dual-index sequencing strategy and curation pipeline for analyzing amplicon sequence data on the MiSeq Illumina sequencing platform. Appl. Environ. Microbiol..

[24] Szuróczki S., Szabó A., Korponai K., Felföldi T., Somogyi B., Márialigeti K., Tóth E. (2020). Prokaryotic community composition in a great shallow soda lake covered by large reed stands (Neusiedler See/Lake Fertő) as revealed by cultivation-and DNA-based analyses. FEMS Microbiol. Ecol..

[25] Schloss P.D. (2009). A high-throughput DNA sequence aligner for microbial ecology studies. PLoS ONE.

[26] Quast C., Pruesse E., Yilmaz P., Gerken J., Schweer T., Yarza P., Peplies J., Glöckner F.O. (2012). The SILVA ribosomal RNA gene database project: Improved data processing and web-based tools. Nucleic Acids Res..

[27] Tindall B.J., Rosselló-Móra R., Busse H.J., Ludwig W., Kämpfer P. (2010). Notes on the characterization of prokaryote strains for taxonomic purposes. Int. J. Syst. Evol. Microbiol..

[28] Szuróczki S., Kéki Z., Káli S., Lippai A., Márialigeti K., Tóth E. (2016). Microbiological investigations on the water of a thermal bath at Budapest. Acta Microbiol. Immunol. Hung..

[29] Kalwasińska A., Felfoeldi T., Walczak M., Kosobucki P. (2015). Physiology and molecular phylogeny of bacteria isolated from alkaline distillery lime. Pol. J. Microbiol..

[30] Yoon S.H., Ha S.M., Kwon S., Lim J., Kim Y., Seo H., Chun J. (2017). Introducing EzBioCloud: A taxonomically united database of 16S rRNA gene sequences and whole-genome assemblies. Int. J. Syst. Evol. Microbiol..

[31] Scorzoni L., Sangalli-Leite F., de Lacorte Singulani J., Costa-Orlandi C.B., Fusco-Almeida A.M., Mendes-Giannini M.J.S. (2016). Searching new antifungals: The use of in vitro and in vivo methods for evaluation of natural compounds. J. Microbiol. Methods.

[32] Tóth E., Szuróczki S., Kéki Z., Kosztik J., Makk J., Bóka K., Spröer C., Márialigeti K., Schumann P. (2017). Brevundimonas balnearis sp. nov., isolated from the well water of a thermal bath. Int. J. Syst. Evol. Microbiol..

[33] Porra R.J., Thompson W.A., Kriedemann P.E. (1989). Determination of accurate extinction coefficients and simultaneous equations for assaying chlorophylls a and b extracted with four different solvents: Verification of the concentration of chlorophyll standards by atomic absorption spectroscopy. Biochim. Biophys. Acta.

[34] Aleström P., D’Angelo L., Midtlyng P.J., Schorderet D.F., Schulte-Merker S., Sohm F., Warner S. (2020). Zebrafish: Housing and husbandry recommendations. Lab. Anim..

[35] Westerfield M. (2000). The Zebrafish Book: A Guide for the Laboratory Use of Zebrafish (Danio Rerio).

[36] Schindelin J., Arganda-Carreras I., Frise E., Kaynig V., Longair M., Pietzsch T., Preibisch S., Rueden C., Saalfeld S., Schmid B. (2012). Fiji: An open-source platform for biological-image analysis. Nat. Methods.

[37] (1998). Act XXVIII of 1998 on the Protection and Humane Treatment of Animals.

[38] (2010). Directive 2010/63/EU of the European Parliament and of the Council of 22 September 2010 on the Protection of Animals Used for Scientific Purposes.

[39] Oksanen J., Kindt R., Legendre P., O’Hara B., Stevens M.H.H., Oksanen M.J., Suggests M.A.S.S. (2007). The vegan package. Community Ecol. Package.

[40] Hammer Ø., Harper D.A.T., Ryan P.D. (2001). PAST: Paleontological statistics software package for education and data analysis. Palaeontol. Electron..

[41] Chen B., Pan Y., Chen Y., Zhang Z., Yang Z., Zheng M., Lu T., Jiang L., Qian H. (2022). TiO_2_ nanoparticles exert an adverse effect on aquatic microbial communities. Sci. Total Environ..

[42] Mohammed Sadiq I., Chandrasekaran N., Mukherjee A.J.C.N. (2010). Studies on effect of TiO_2_ nanoparticles on growth and membrane permeability of Escherichia coli, Pseudomonas aeruginosa, and *Bacillus subtilis*. Curr. Nanosci..

[43] Ammendolia M.G., Iosi F., De Berardis B., Guccione G., Superti F., Conte M.P., Longhi C. (2014). Listeria monocytogenes behaviour in presence of non-UV-irradiated titanium dioxide nanoparticles. PLoS ONE.

[44] Mathur A., Parashar A., Chandrasekaran N., Mukherjee A. (2017). Nano-TiO_2_ enhances biofilm formation in a bacterial isolate from activated sludge of a waste water treatment plant. Int. Biodeterior. Biodegrad..

[45] Balíková K., Vojtková H., Duborská E., Kim H., Matúš P., Urík M. (2022). Role of exopolysaccharides of Pseudomonas in heavy metal removal and other remediation strategies. Polymers.

[46] Zhang L., Xu L., Graham N., Yu W. (2022). Unraveling membrane fouling induced by chlorinated water versus surface water: Biofouling properties and microbiological investigation. Engineering.

[47] Gudiña E.J., Couto M.R., Silva S.P., Coelho E., Coimbra M.A., Teixeira J.A., Rodrigues L.R. (2022). Sustainable exopolysaccharide production by Rhizobium viscosum CECT908 using corn steep liquor and sugarcane molasses as sole substrates. Polymers.

[48] Wang L., Deng S., Wang S., Su H. (2017). Analysis of aerobic granules under the toxic effect of ampicillin in sequencing batch reactors: Performance and microbial community. J. Environ. Manag..

[49] Van Veen W.L., Van Der Kooij D., Geuze E.C.W.A., Van der Vlies A.W. (1973). Investigations on the sheathed bacterium *Haliscomenobacter hydrossis* gen. n., sp. n., isolated from activated sludge. Antonie Van Leeuwenhoek.

[50] Zhou Z., Jiao C., Liang Y., Du A., Zhang J., Xiong J., Chen G., Zhu H., Lu L. (2022). Study on Degradation of 1,2,4-TrCB by Sugarcane Cellulose-TiO_2_ Carrier in an Intimate Coupling of Photocatalysis and Biodegradation System. Polymers.

[51] Zhu B., Xia X., Xia N., Zhang S., Guo X. (2014). Modification of fatty acids in membranes of bacteria: Implication for an adaptive mechanism to the toxicity of carbon nanotubes. Environ. Sci. Technol..

[52] Kotchaplai P., Khan E., Vangnai A.S. (2017). Membrane alterations in Pseudomonas putida F1 exposed to nanoscale zerovalent iron: Effects of short-term and repetitive nZVI exposure. Environ. Sci. Technol..

[53] Qian J., Li K., Wang P., Wang C., Shen M., Liu J., Lu B., Tian X. (2017). Toxic effects of three crystalline phases of TiO_2_ nanoparticles on extracellular polymeric substances in freshwater biofilms. Bioresour. Technol..

[54] Li J., Yang S., Lei R., Gu W., Qin Y., Ma S., Chen K., Chang Y., Bai X., Xia S. (2018). Oral administration of rutile and anatase TiO_2_ nanoparticles shifts mouse gut microbiota structure. Nanoscale.

[55] Jin C., Tang Y., Yang F.G., Li X.L., Xu S., Fan X.Y., Huang Y.Y., Yang Y.J. (2011). Cellular toxicity of TiO_2_ nanoparticles in anatase and rutile crystal phase. Biol. Trace Elem. Res..

[56] Tong T., Shereef A., Wu J., Binh C.T.T., Kelly J.J., Gaillard J.F., Gray K.A. (2013). Effects of material morphology on the phototoxicity of nano-TiO_2_ to bacteria. Environ. Sci. Technol..

[57] Planchon M., Ferrari R., Guyot F., Gélabert A., Menguy N., Chanéac C., Thill A., Benedetti M.F., Spalla O. (2013). Interaction between Escherichia coli and TiO2 nanoparticles in natural and artificial waters. Colloids Surf. B Biointerfaces.

[58] Lin X., Li J., Ma S., Liu G., Yang K., Tong M., Lin D. (2014). Toxicity of TiO_2_ nanoparticles to *Escherichia coli*: Effects of particle size, crystal phase and water chemistry. PLoS ONE.

[59] Yu Q., Wang H., Peng Q., Li Y., Liu Z., Li M. (2017). Different toxicity of anatase and rutile TiO_2_ nanoparticles on macrophages: Involvement of difference in affinity to proteins and phospholipids. J. Hazard. Mater..

[60] Uboldi C., Urbán P., Gilliland D., Bajak E., Valsami-Jones E., Ponti J., Rossi F. (2016). Role of the crystalline form of titanium dioxide nanoparticles: Rutile, and not anatase, induces toxic effects in Balb/3T3 mouse fibroblasts. Toxicol. In Vitro.

[61] Iswarya V., Bhuvaneshwari M., Alex S.A., Iyer S., Chaudhuri G., Chandrasekaran P.T., Bhalerao G.M., Chakravarty S., Raichur A.M., Chandrasekaran N. (2015). Combined toxicity of two crystalline phases (anatase and rutile) of Titania nanoparticles towards freshwater microalgae: *Chlorella* sp. Aquat. Toxicol..

[62] Gerloff K., Fenoglio I., Carella E., Kolling J., Albrecht C., Boots A.W., Förster I., Schins R.P.F. (2012). Distinctive toxicity of TiO_2_ rutile/anatase mixed phase nanoparticles on Caco-2 cells. Chem. Res. Toxicol..

[63] Song U., Jun H., Waldman B., Roh J., Kim Y., Yi J., Lee E.J. (2013). Functional analyses of nanoparticle toxicity: A comparative study of the effects of TiO_2_ and Ag on tomatoes (*Lycopersicon esculentum*). Ecotoxicol. Environ. Saf..

[64] Du W., Sun Y., Ji R., Zhu J., Wu J., Guo H. (2011). TiO_2_ and ZnO nanoparticles negatively affect wheat growth and soil enzyme activities in agricultural soil. J. Environ. Monit..

[65] Jacob D.L., Borchardt J.D., Navaratnam L., Otte M.L., Bezbaruah A.N. (2013). Uptake and translocation of Ti from nanoparticles in crops and wetland plants. Int. J. Phytoremediation.

[66] Kelemen G., Keresztes A., Bacsy E., Feher M., Fodor P., Pais I. (1993). Distribution and intracellular localization of titanium in plants after titanium treatment. Food Struct..

[67] Dietz K.J., Herth S. (2011). Plant nanotoxicology. Trends Plant Sci..

[68] Bar-Ilan O., Louis K.M., Yang S.P., Pedersen J.A., Hamers R.J., Peterson R.E., Heideman W. (2012). Titanium dioxide nanoparticles produce phototoxicity in the developing zebrafish. Nanotoxicology.

[69] Wang Y.J., He Z.Z., Fang Y.W., Xu Y., Chen Y.N., Wang G.Q., Yang Y.Q., Yang Z., Li Y.H. (2014). Effect of titanium dioxide nanoparticles on zebrafish embryos and developing retina. Int. J. Ophthalmol..

[70] Tang T., Zhang Z., Zhu X. (2019). Toxic effects of TiO_2_ NPs on zebrafish. Int. J. Environ. Res. Public Health.

[71] Samaee S.M., Rabbani S., Jovanović B., Mohajeri-Tehrani M.R., Haghpanah V. (2015). Efficacy of the hatching event in assessing the embryo toxicity of the nanosized TiO_2_ particles in zebrafish: A comparison between two different classes of hatching-derived variables. Ecotoxicol. Environ. Saf..

[72] Chen Z., Wang Y., Ba T., Li Y., Pu J., Chen T., Song Y., Gu Y., Qian Q., Yang J. (2014). Genotoxic evaluation of titanium dioxide nanoparticles in vivo and in vitro. Toxicol. Lett..

[73] Armand L., Tarantini A., Beal D., Biola-Clier M., Bobyk L., Sorieul S., Pernet-Gallay K., Marie-Desvergne C., Lynch I., Herlin-Boime N. (2016). Long-term exposure of A549 cells to titanium dioxide nanoparticles induces DNA damage and sensitizes cells towards genotoxic agents. Nanotoxicology.

[74] Wallin H., Kyjovska Z.O., Poulsen S.S., Jacobsen N.R., Saber A.T., Bengtson S., Jackson P., Vogel U. (2017). Surface modification does not influence the genotoxic and inflammatory effects of TiO_2_ nanoparticles after pulmonary exposure by instillation in mice. Mutagenesis.

[75] Botelho M.C., Costa C., Silva S., Costa S., Dhawan A., Oliveira P.A., Teixeira J.P. (2014). Effects of titanium dioxide nanoparticles in human gastric epithelial cells in vitro. Biomed. Pharmacother..

[76] Bhattacharya K., Davoren M., Boertz J., Schins R.P., Hoffmann E., Dopp E. (2009). Titanium dioxide nanoparticles induce oxidative stress and DNA-adduct formation but not DNA-breakage in human lung cells. Part. Fibre Toxicol..

[77] Dorier M., Brun E., Veronesi G., Barreau F., Pernet-Gallay K., Desvergne C., Rabilloud T., Carapito C., Herlin-Boime N., Carrière M. (2015). Impact of anatase and rutile titanium dioxide nanoparticles on uptake carriers and efflux pumps in Caco-2 gut epithelial cells. Nanoscale.

[78] Bettini S., Boutet-Robinet E., Cartier C., Coméra C., Gaultier E., Dupuy J., Naud N., Taché S., Grysan P., Reguer S. (2017). Food-grade TiO_2_ impairs intestinal and systemic immune homeostasis, initiates preneoplastic lesions and promotes aberrant crypt development in the rat colon. Sci. Rep..

[79] Butler K.S., Casey B.J., Garborcauskas G.V., Dair B.J., Elespuru R.K. (2014). Assessment of titanium dioxide nanoparticle effects in bacteria: Association, uptake, mutagenicity, co-mutagenicity and DNA repair inhibition. Mutat. Res. Genet. Toxicol. Environ. Mutagen..

[80] Nam S.H., Kim S.W., An Y.J. (2013). No evidence of the genotoxic potential of gold, silver, zinc oxide and titanium dioxide nanoparticles in the SOS chromotest. J. Appl. Toxicol..

[81] Alhadrami H.A., Shoudri R.A. (2021). Titanium Oxide (TiO_2_) Nanoparticles for Treatment of Wound Infection. J. Pure Appl. Microbiol..

