# Supplementary material for: System-Dependent Ecotoxicological Effects of Anatase and Rutile Titanium Dioxide Nanoparticles Across Prokaryotic and Eukaryotic Test Models
Source: Nanomaterials (Basel). 2026 Jul 10;16(14):849. doi: 10.3390/nano16140849 (PMC13415024; doi:10.3390/nano16140849)
Supplement: Supplementary file 1 [file nanomaterials-16-00849-s001.zip › nanomaterials-4384176-supplementary.pdf]

Supplementary Materials

System-Dependent Ecotoxicological Effects of Anatase and Rutile Titanium Dioxide Nanoparticles Across Prokaryotic and Eukaryotic Test Models

Gergely Krett [1,*], Rózsa Farkas [1], Máté Varga [2], Tamás Annus [2], Linda Marzougui [3], Ádám Solti [3], Károly Bóka [4] and Erika Tóth [1,5,*]

1 Department of Microbiology, Eötvös Loránd University, Pázmány Péter Lane 1/c, 1117 Budapest, Hungary
2 Department of Genetics, Eötvös Loránd University, Pázmány Péter Lane 1/c, 1117 Budapest, Hungary
3 Department of Plant Physiology and Molecular Plant Biology, Eötvös Loránd University, Pázmány Péter Lane 1/c, 1117 Budapest, Hungary; adam.solti@ttk.elte.hu (Á.S.)
4 Department of Plant Anatomy, Eötvös Loránd University, Pázmány Péter Lane 1/c, 1117 Budapest, Hungary; karoly.boka@ttk.elte.hu
5 Research and Development Center, Eszterházy Károly Catholic University, Leányka Str. 8/G, 3300 Eger, Hungary
* Correspondence: gergely.krett@ttk.elte.hu (G.K.); erika.toth@ttk.elte.hu (E.T.)

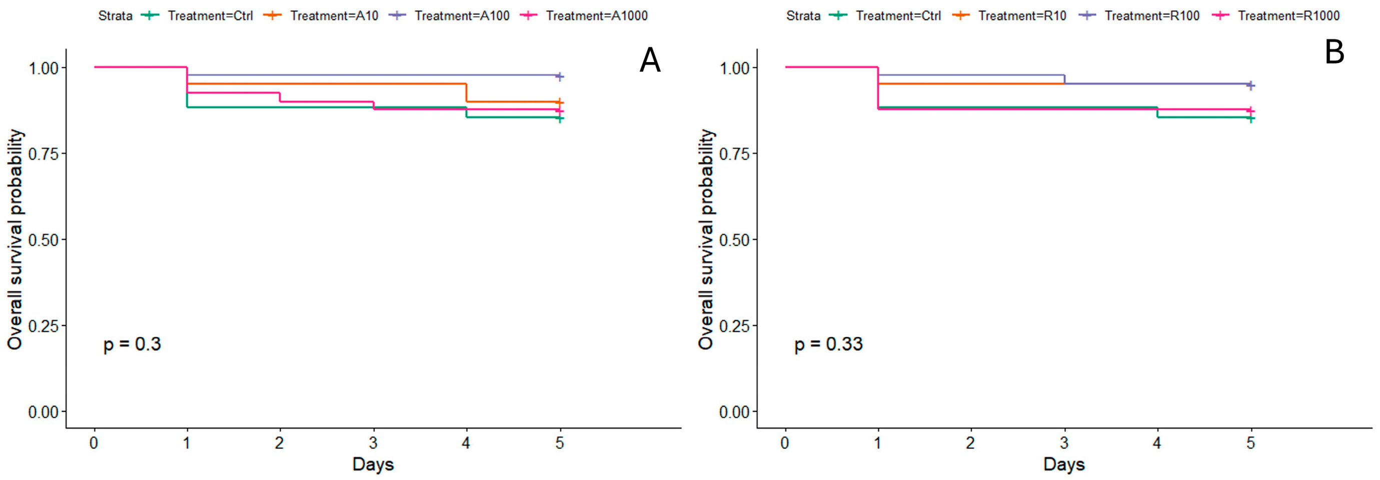

Figure S1. Overall survival probability of zebrafish embryos treated with different concentrations of nano-TiO_2 (A: anatase, B: rutile, Ctrl: control).

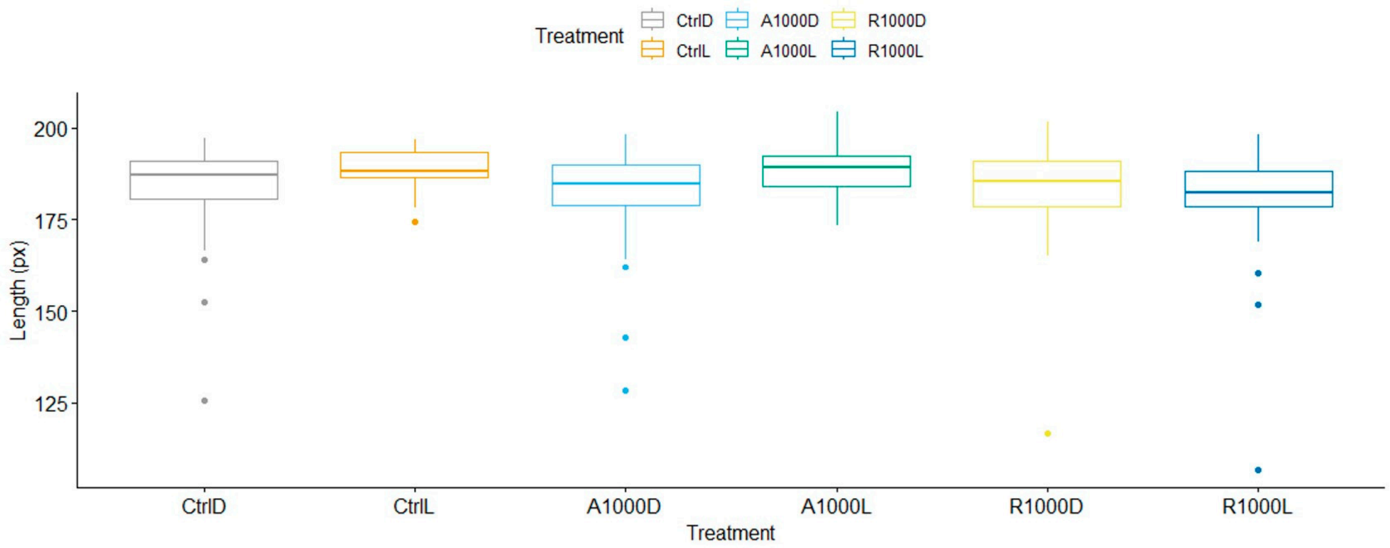

Figure S2. Total length of zebrafish embryos treated with different concentrations of nano-TiO_2 in the absence of light measured at 96 hours post-fertilization (A: anatase, B: rutile, Ctrl: control, D: dark, L: light).

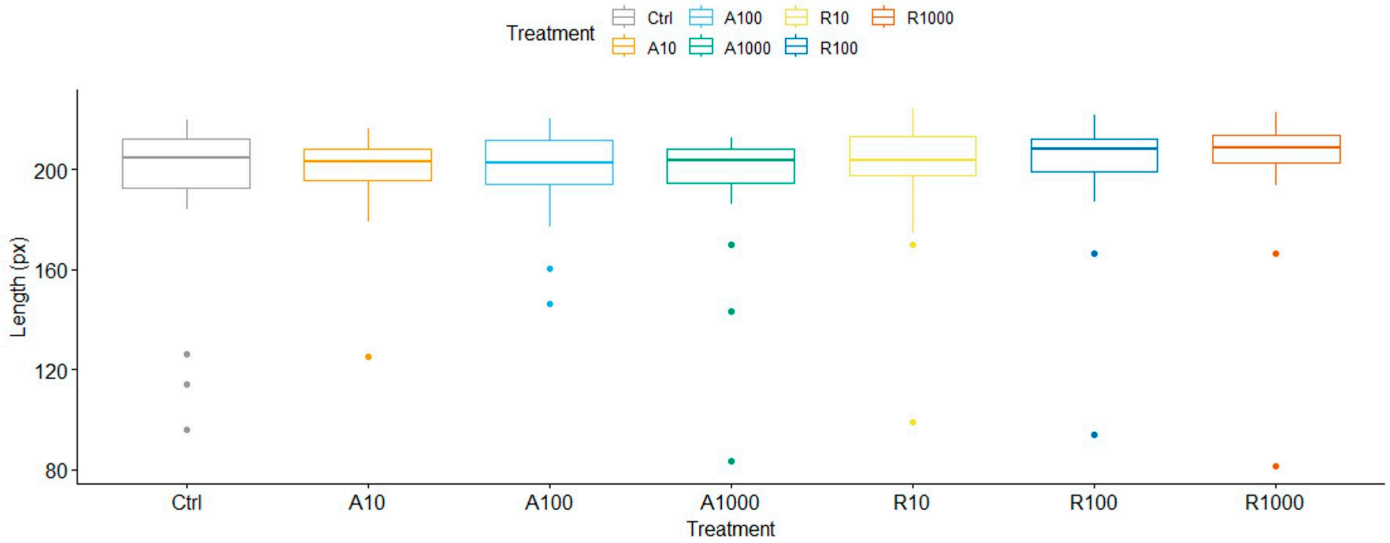

Figure S3. Total length of zebrafish embryos treated with different concentrations of nano-TiO_2 in the presence of sunlight measured at 5 days post-fertilization (A: anatase, R: rutile, Ctrl: control).

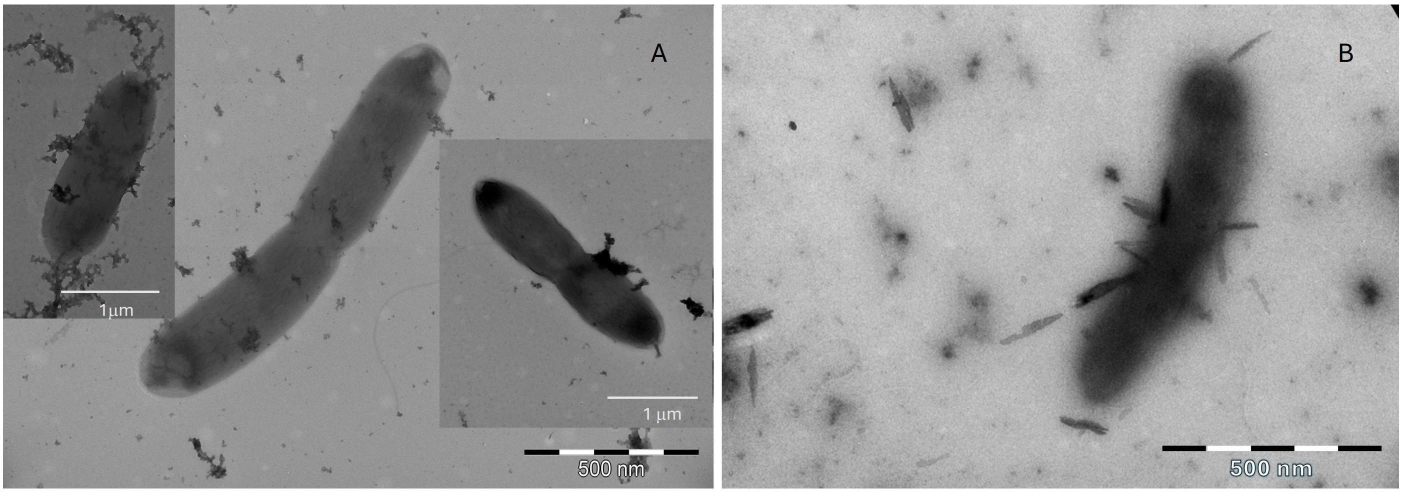

Figure S4. Transmission electron microscopy images of *Pseudomonas vancouverensis* strain T5 after treatment with 500 ppm anatase (A) and rutile (B) TiO_2 nanoparticles. TiO_2 nanoparticles were observed in close association with the bacterial cell surface.
